# The Physical Position of Man in Creation

**Published:** 1845-05

**Authors:** 


*The physical position of man in creation.*—The law of unity of type and function in animals, applied in the preceding pages to the function of the cerebro-spinal axis in man, has shown (what is necessarily deduced from the law itself) that the transition of structure and function is gradual, and consequently, no strong line of demarcation can be drawn between the manifestations of its various functions. The automatic acts pass insensibly into the reflex, the reflex into the instinctive, the instinctive are *quasi* emotional, the emotional are intellectual. This gradation of structure and function observed in the nervous system, is observed also with reference to all other structures of his body. Man is at the head of a vast ascending scale of animal life, so extended in its connexions downwards, that for the present purpose it may be considered as infinitely extended. With our existing knowledge of of the uniformity of the laws of creation, the deduction is absolutely incontrovertible, the scale of being is not truncated at man, and that beyond him there cannot be a dark, unpeopled void. The law of gradation of development vigorously pushed to its legitimate conclusions points out an infinite gradation of being *above* and *superior* to man. That we cannot see such beings, nor demonstrate their existence is a necessary result of our position in the scale, and no proof whatever of their nonexistence. The worm knows nothing of man, his works or his actions: nothing of the sun or the stars, of the beings swarming around it; and so with reference to the spiritual world, the world around and above *us*—our organs may be and doubtless are, as imperfect as those of the worm with reference to the world around and above *it*. Man is then at the foot of another scale of beings, the highest of which *at least*, as far transcends man, as man transcends the zoophyte. This proposition, I repeat, is the unavoidable inference from our present physiological knowledge, and is a complete answer to those good, zealous, but not wise men, who think science leads to scepticism and irreligion. It leads to a rational faith utterly opposed to arrogant infidelity.—*Dr Laycock in Brit. & For. Med. Reveiw.*

